# Supplementary material for: Tuning the Interactions in Multiresponsive Complex Coacervate-Based Underwater Adhesives
Source: Int J Mol Sci. 2019 Dec 21;21(1):100. doi: 10.3390/ijms21010100 (PMC6982270; doi:10.3390/ijms21010100)
Supplement: Supplementary file 1 [file ijms-21-00100-s001.pdf]

International Journal of *Molecular Sciences*

Supporting Information

Tuning the Interactions in Multiresponsive Complex Coacervate-Based Underwater Adhesives

Marco Dompé [1], Francisco J. Cedano-Serrano [2], Mehdi Vahdati [2], Ugo Sidoli [3], Olaf Heckert [1], Alla Synytska [3], Dominique Hourdet [2], Costantino Creton [2], Jasper van der Gucht [1], Thomas Kodger [1] and Marleen Kamperman [1,4,*]

[1] Laboratory of Physical Chemistry and Soft Matter, Wageningen University & Research, Stippeneng 4, 6708 WE Wageningen, The Netherlands; marco.dompe@wur.nl (M.D.); olaf.heckert@wur.nl (O.A.); jasper.vandergucht@wur.nl (J.v.d.G.); thomas.kodger@wur.nl (T.K.); marleen.kamperman@rug.nl (M.K.)

[2] Soft Matter Sciences and Engineering, ESPCI Paris, PSL University, Sorbonne University, CNRS, F-75005, Rue Vauquelin 10, Paris, France; francisco.cedano@espci.fr (F.J.C.S.); mehdi.vahdati@espci.fr (M.V.); dominique.hourdet@espci.fr (D.H.); costantino.creton@espci.fr (C.C.)

[3] Leibniz-Institut für Polymerforschung Dresden e.V., Hohe Straße 6, 01069 Dresden, Germany; sidoli@ipfdd.de (U.S.); synytska@ipfdd.de (A.S.)

[4] Laboratory of Polymer Science, Zernike Institute for Advanced Materials, University of Groningen, Nijenborgh 4, 9747 AG Groningen, The Netherlands; marleen.kamperman@rug.nl (M.K.)

* Correspondence: marleen.kamperman@rug.nl;

Keywords: complex coacervate, poly(*N*-isopropylacrylamide), polyelectrolytes, underwater adhesion, environmentally-triggered setting process, LCST, non-covalent interactions, bioinspired materials

1. Water Content

In Figure S1, the water content is reported as function of PNIPAM content in complex coacervates.

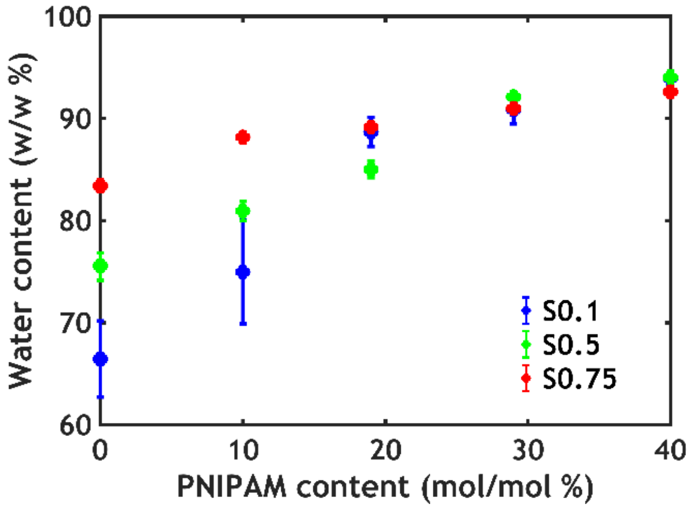

Figure S1 Water content as function of PNIPAM content

2. Rheology

In Figure S2 the complex viscosity recorded as a function of angular frequency is reported for the sample P40S0.75.

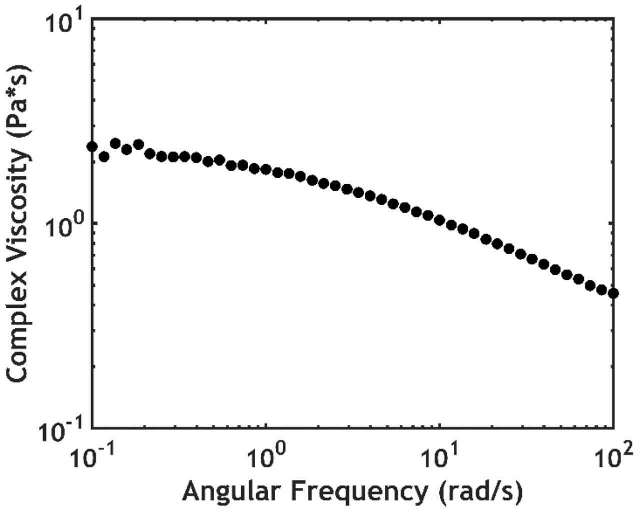

Figure S2 Complex viscosity as function of angular frequency for P40S0.75

The values reported in Figure 2A are the ones recorded, for every analysed sample, at the lowest frequency, where a Newtonian plateau is observed: in this region the values approach the zero shear viscosity.

By lowering the ionic strength, the onset of the transition shifts to higher temperatures, in agreement with the DSC data previously recorded (Figure S3A). In addition to that, the strengthening effect become less and less marked: the complex coacervate is already a solid gel at 20 °C, so that no crossover is obviously visible.

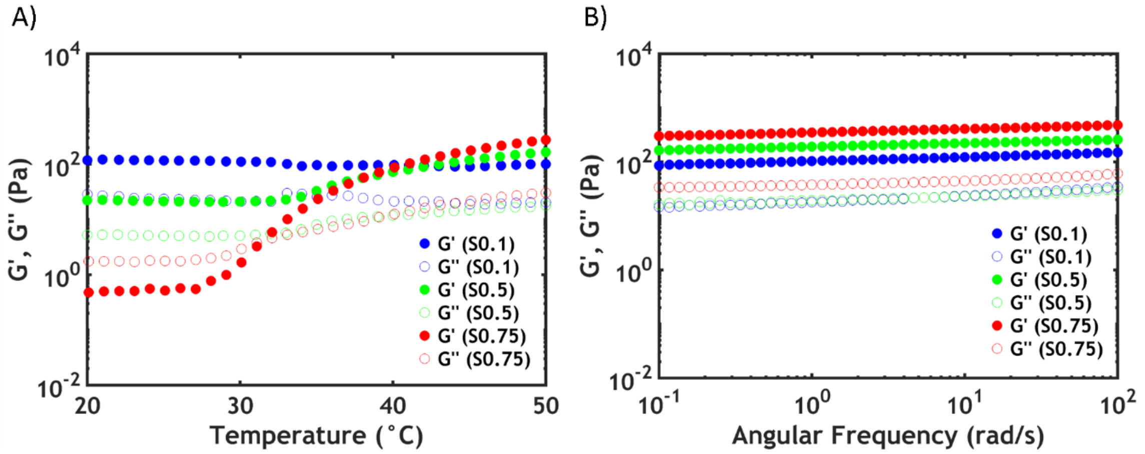

Figure S3 A) Temperature sweeps and **B)** frequency sweeps performed at 50 °C as function of [NaCl] for P40Sy.

At 50 °C, when lowering the ionic strength, the moduli slightly decrease: this means that the strengthening mechanism is more effective when the transition occurs at high salt concentration (Figure S3B). This might be due to the mobility of the PNIPAM chains. At 0.75 M NaCl the complex coacervate is initially a liquid and the interactions between the polyelectrolyte components are weak: the PNIPAM units can therefore easily aggregate, forming well-entangled nodes. However, at lower ionic strength, the interactions between the charged components are much stronger: when raising the temperature, the PNIPAM chains do not have the required mobility to find each other so that a huge increase in moduli is not observed.

3. Underwater Adhesion

In Figure S4, the stress-strain curves obtained after a temperature switch are plotted in log-lin scale.

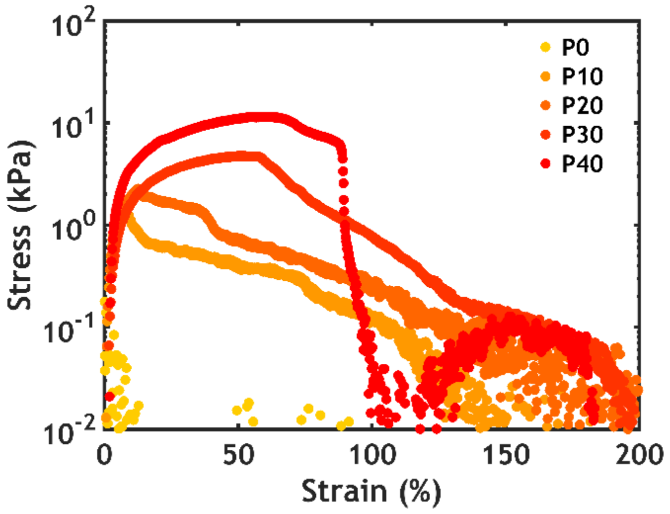

Figure S4 Stress-strain curves plotted in log-lin scale.

When PNIPAM is present, small filaments are formed which help to sustain more stress at higher strain and which break leaving residues on the detaching probe.

4. ^{1}H-NMR spectra of graft copolymers

PAA-*g*-PNIPAM30 (Figure S5): PAA (^{1}H-NMR, 400 MHz, D_2O, δ (ppm)): 1.46-1.68 (2H,CH_2 backbone), 2.10 (1H, CH backbone). PNIPAM (^{1}H-NMR, 400 MHz, D_2O, δ (ppm)): 1.14 (6H, CH_3), 1.58 (2H, CH_2 backbone), 2.02 (1H, CH backbone), 3.88 (1H, CH).

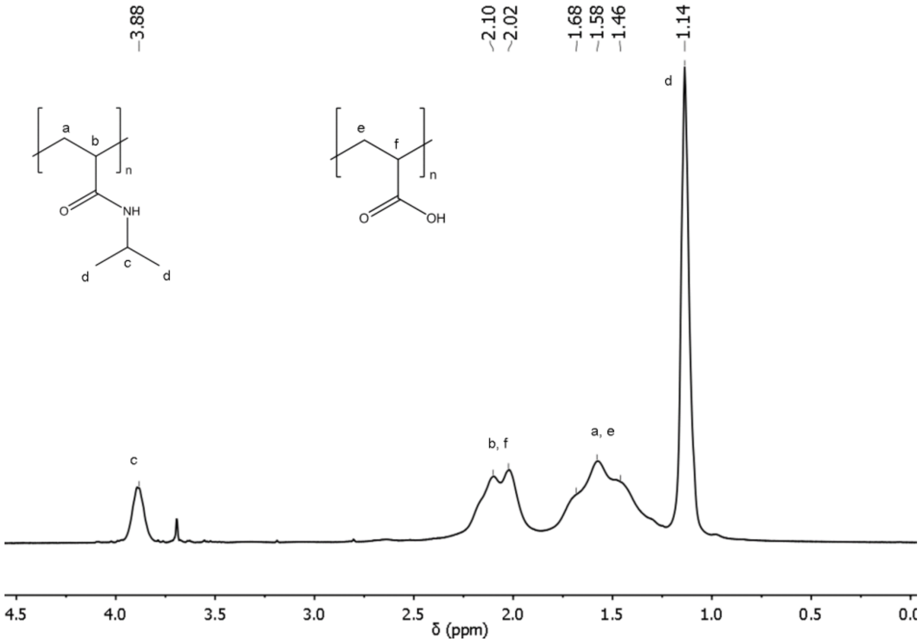

Figure S5 ^{1}H-NMR spectrum of PAA-*g*-PNIPAM.

The molar ratio of PNIPAM sidechains was determined as follows. At first, the area of the peak at 3.88 ppm was set to 1.0. Afterwards, in order to get the PAA contribution to the ^{1}H-NMR spectrum, the area between 1.25 ppm and 2.5 ppm was subtracted by 3.0 (number of hydrogens belonging to the PNIPAM backbone) and successively divided by 3.0 (number of hydrogens belonging to the PAA backbone). The molar ratio of PNIPAM sidechains was then obtained by dividing the area relative to one PNIPAM hydrogen (1.0) by the sum of the areas relative to one PAA hydrogen and one PNIPAM hydrogen.

MacroPNIPAM (Figure S2): (^{1}H-NMR, 400 MHz, D_2O (solvent peak at 4.70), δ (ppm)): 1.14 (6H, CH_3), 1.58 (2H, CH_2 backbone), 2.01 (1H, CH backbone), 2.63 (2H, CH_2), 3.45 (2H, CH_2), 3.90 (1H, CH), 5.77, 6.20 and 6.38 (3H, end-group).

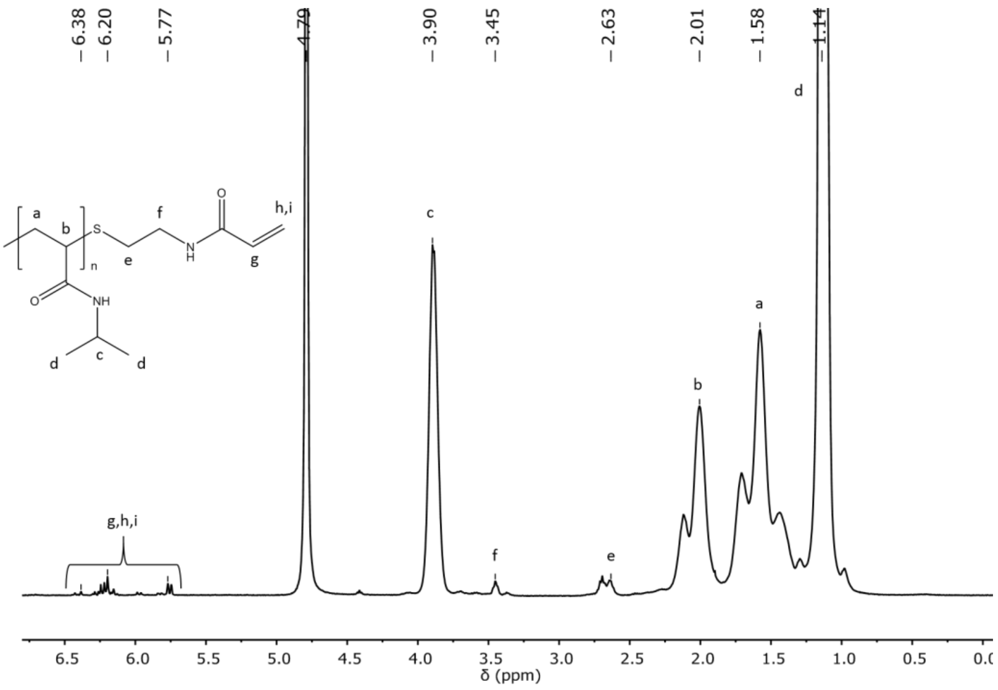

Figure S6 ^{1}H-NMR spectrum of macroPNIPAM.

In Figure S6 it is possible to detect the presence of the double bond in the region between 5.5 ppm and 6.5 ppm, which means that the coupling reaction has been performed successfully.

PDMAPAA-*g*-PNIPAM30 (Figure S7): PDMAPAA (^{1}H-NMR, 400 MHz, D_2O, δ (ppm)): 1.63 (1H, CH backbone), 1.79 (2H, CH_2), 2.08 (1H, CH backbone), 2.41 (6H, CH_3), 2.59 (2H, CH_2), 3.22 (2H, CH_2). PNIPAM (^{1}H-NMR, 400 MHz, D_2O, δ (ppm)): 1.19 (6H, CH_3), 1.63 (2H, CH_2 backbone), 2.08 (1H, CH backbone), 3.95 (1H, CH).

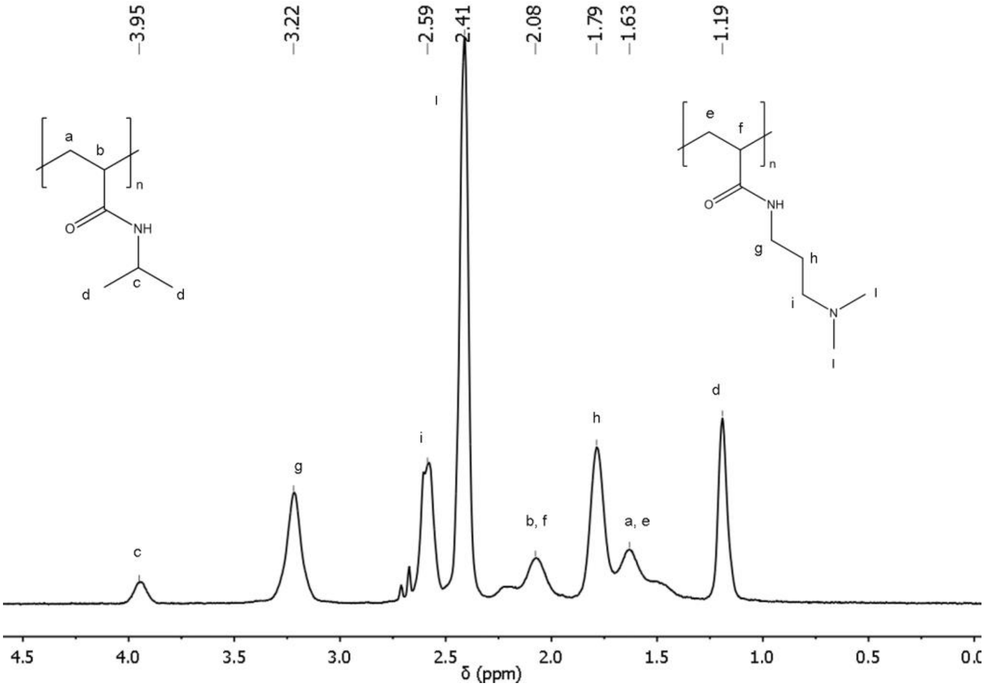

Figure S7 ^{1}H-NMR spectrum of PDMAPAA-*g*-PNIPAM.

The mol% of PNIPAM sidechains was determined as follows. At first, the area of the peak at 1.19 ppm, corresponding to 6 hydrogens in the PNIPAM isopropyl group, was set to 1.0. Afterwards, the area of the peak at 2.41, corresponding to 6 hydrogens in the PDMAPAA dimethylamine group, was determined. The molar ratio of PNIPAM sidechains was then obtained by calculating the ratio between the PNIPAM signal (1.0) and the sum of the PNIPAM and PDMAPAA signals.
